# Individual Differences in the Adoption of Sound Change

**DOI:** 10.1177/0023830920959753

**Published:** 2020-10-25

**Authors:** Cesko C. Voeten

**Affiliations:** Leiden Institute for Brain and Cognition, Leiden University Centre for Linguistics, Netherlands

**Keywords:** Sound change, individual differences, sociophonetics, perception and production, laboratory phonology

## Abstract

It is still unclear whether an individual’s adoption of on-going sound change starts in production or in perception, and what the time course of the adoption of sound change is in adult speakers. These issues are investigated by means of a large-scale (106 participants) laboratory study of an on-going vowel shift in Dutch. The shift involves the tense mid vowels /eː,øː,oː/, which are changing into phonologically conditioned upgliding diphthongs, and the original diphthongs /εi,œy,ɔu/, whose nuclei are lowering. These changes are regionally stratified: they have all but completed in the Netherlands, but have not affected the variety of Dutch spoken in neighboring Belgium. The study compares production (word-list reading) and perception (rhyme decision) data from control groups from each country to those of 18 “sociolinguistic migrants”: Belgian individuals who moved to the Netherlands years ago. Data are analyzed using mixed-effects models, considering not just the group level, but also individual differences. Production results show that at the group level, the migrant group is in between the two control groups, but at the individual level it becomes apparent that some migrants have adopted the Netherlandic norms, but others have not. Perception results are similar to the production results at the group level. Individual-level results do not provide a clear picture for the perception data, but the individual differences in perception correlate with those in production. The results agree with and extend previous findings on the role of individual differences in the individual adoption and eventual community propagation of on-going sound change.

## 1 Introduction

### 1.1 The adoption of sound change

It has been suggested that sound change originates when there is a mismatch (or “coordination failure”; Bermúdez-Otero, 2007) between a speaker and a listener. Either they have the same grammar, but one of the pair over- or underapplies rules compensating for intrinsic variation (Ohala, 1981), or the speaker and the listener have acquired subtly different grammars in childhood and assign different cue weights to the same auditory information in the phonetic signal (Beddor, 2009; Hamann, 2009). When the originating individual begins to reproduce their individual grammatical innovation, and hence begins to transmit the sound change to other individuals through the medium of speech production, the sound change is considered to have been actuated. After that point, it will either spread to other members of the community or peter out. The prerequisites for a sound change to originate, actuate, and spread are thus exceedingly rare: one needs a specific type of variation which is conducive to coordination failure at the right place at the right time, one or more specific individuals to initiate a sound change based on this variation, and a specific reception by the speech community (namely one in which the change is copied and again transmitted further). The rarity of this specific combination of individual and community characteristics has been considered both the reason why sound change takes place at all (if such eventualities were commonplace, we would have learned to be robust against them), and is rare to actuate in the first place (Stevens & Harrington, 2014).

Following Pinget (2015), we may assume that sound change spreads through a continuous chain of actuations by individual speaker–listener interactions. The present paper focuses on the individuals who form the links of this chain: how do they adopt an actuated sound change from their interlocutor into their own grammars? This question is positioned squarely in between the issues of actuation and community spread. It is related to Weinreich et al.’s (1968) transmission problem, but in an individualized form; the transmission problem by Weinreich et al. (1968) concerns the dissemination of sound change throughout the community (or its grammar) as a whole. The idea that sound change needs to be initiated by individuals follows from classic models of origin and actuation by authors such as Ohala (1981) and Hyman (1976) (who have since also refined their positions to incorporate representations of the phonetic implementation; Hyman, 2013; Ohala, 2012), and is also fully compatible with the model by Beddor (2009). It is *almost* compatible with the model by Hamann (2009); her model differs from that by Beddor (2009) in the aspect that, like Labov (2007), Hamann posits that the crucial phonological reanalysis can only be made by children, and that adults can only perform superficial phonetic reanalysis. These models can be considered the theoretical-phonological backdrop of this paper.

### 1.2 Perception, production, and the individual

Recent work on sound change has recognized that the adoption of sound change by individual speakers and listeners relies on the link between their perception and their production. Consider, for instance, Baker et al. (2011), who study American English [s]-retraction in words like “street.” Their results show that speaker-specific coarticulatory variation between the [s] and the following [ɹ] leads to inter-speaker variation in the degree of [s]-retraction. Speakers who strongly coarticulate the [s] with the following [ɹ] produce an [s] that is realized similarly to [ʃ]. For speakers who coarticulate only weakly, that sound is considered a distinct articulatory target. When the latter speakers are paired as listeners with strong [s]-retractors, the weak retractors have an opportunity to actuate a sound change, if their percept of [s] as [ʃ] is reanalyzed to /ʃ/ (or to an equivalent phonological rule) and they begin to use that system in their own productions. Beddor et al. (2018) found that the link between production and perception extends also to the *time course* according to which listeners make use of phonetic cues. Their results show that participants’ production of coarticulatory nasalization was predictive of the time course of their perception of the same information. As the authors note, this suggests that differences in perception grammars need not be restricted to cue weightings *per se* (cf. Beddor, 2009; Hamann, 2009), but can also lie in which cues are utilized when. The results by Beddor et al. (2018) also show that this perception–production link remains stable during on-going sound change—that is, participants who are more advanced along a sound change in perception are also more advanced in production, causing them to spread the sound change further.

There is evidence that the roles of perception and production reverse depending on the degree to which a sound change has progressed at the community level. Pinget et al. (2019) (also in Pinget, 2015) show that, for the on-going merger of Dutch /v,z,ɣ/ into /f,s,x/ and the incipient merger of Dutch /b/ into /p/, adoption by individuals starts in perception: one needs to perceive a change before one will produce it. However, as the change progresses, this relationship slowly comes to reverse: an individual who does not produce a contrast anymore may still be able to draw on subtle differences in perception (although cf. Labov et al.’s [1972] “near-mergers”). Coetzee et al. (2018) make a similar observation concerning tonogenesis in Afrikaans: while they generally find that speakers’ use of VOT versus F0 in the production of phonologically voiced versus voiceless plosives correlates with their use in perception, four of their participants did not produce a reliable VOT contrast, but did rely on such cues in perception. Importantly, the reverse was not found, corroborating Pinget et al. (2019) that, for incipient changes, perception precedes production.

Besides formal linguistic variation such as differences in production and perception grammars, there is evidence that variation at the individual level plays a role in the extent to which sound change is actuated and propagated. Studies of this aspect of sound change largely reinforce the stereotype that leaders of change are young, educated women with certain personality attributes (Haeri, 1991; Labov, 2001; Milroy, 1993; Yu, 2010, 2013) and large social networks (Denis, 2011; Lev-Ari, 2018). These characteristics overlap with the individuals who fit Marslen-Wilson’s (1973) description of “close shadowers” in speech-shadowing tasks, which suggests that these personality factors are not directly responsible, but rather indirectly affect socio-cognitive processing (see Yu, 2013), and that the latter is what causes these individuals to be leaders of sound change as well.

The role of individual-level factors, and hence of all of the factors discussed in this section, may also depend on whether a sound change is system-internal or contact-driven.^1^ The theories by Beddor (2009), Hamann (2009), and Ohala (1981) are mainly concerned with system-internal changes, such as those in Coetzee et al. (2018) or Pinget (2015). Following Hamann (2009) and Labov (2007), these changes spread via L1 acquisition. In contrast, contact-driven changes are spread via contact between adult speakers and listeners. The most obvious factor affecting adults’ adoption of contact-driven changes is the amount of time that they have been exposed to the sound changes. Generally speaking, the shorter the timespan, the more heterogeneous individuals are in adopting an ambient phonetic change. This is illustrated by Alshangiti and Evans (2011), Bauer (1985), Carter (2007), Cedergren (1987), Chambers (1992), De Decker (2006), Evans and Iverson (2007), Harrington (2006, 2007), Harrington et al. (2000, 2005), Hinton (2015), Nahkola and Saanilahti (2004), Nycz (2011, 2013), van Oostendorp (2008), Prince (1987), Sankoff (2004), Sankoff and Blondeau (2007), Sankoff et al. (2001), Trudgill (1988), Wagner (2008), Yaeger-Dror (1994), and Ziliak (2012), who all found small adoption effects in small minorities of studied individuals. When such changes become fully stable within a community is not precisely known, although it has been shown that 15 years can be enough (Gordon & Maclagan, 2001; Trudgill, 1999; Yaeger-Dror, 1994). The speed with which a change is spread likely depends on its salience (Auer et al., 1998; see e.g., Rácz, 2013 for what this could mean) to the listener: individuals who are more attentive to a change in perception are more likely to adopt the change in production, particularly if the change involves social indexation (Beddor, 2015).

### 1.3 Phonological change versus phonetic change

The degree to which a sound change, particularly one that is contact-induced, can be adopted by individuals depends on the type of the sound change. According to Labov (2007) and Hamann (2009), phonological reanalyses are restricted to language-learning infants, with adult speakers only being able to enact superficial phonetic changes. In the case of phonological change, the sound change involves the phonological grammar, either by adding or deleting a phonological rule, or by adding, removing, or substituting a phonemic category. A clear example of such a case is offered by Harrington et al. (2008), who studied /uː/-fronting in Standard Southern British English (henceforth: SSBE). In an apparent-time study of younger and older SSBE participants, they found that the younger group had a more fronted /uː/ category both in production (i.e., [ʉː]) and in perception (i.e., /ʉː/). Since Harrington et al. (2008) show that the perception change preceded the production change, they conclude that SSBE /uː/-fronting started with a phonological change in the underlying form (/uː/>/ʉː/). In a different study, Kleber et al. (2012) show that the same account holds for SSBE /ʊ/-fronting, except that this change is in an earlier stage of completion.

In contrast to phonological changes, some changes within the grammars of individuals do not involve a representational change but merely change the phonetic implementation of a particular segment. Such changes are reported by, for instance, Evans and Iverson (2007), who followed 19 British-English high-school students who were about to enter university. After two years, these students’ vowel systems had become more aligned with SSBE in production, but no reliable change was found in perception. The lack of reliable findings in perception suggests that only the phonetic implementation has changed, and not the phonological representation. It also shows that, for these changes, production changed before perception did, in contrast to the aforementioned results. A possible related observation is that, as pointed out by an anonymous reviewer, one of the changes studied by Evans and Iverson (2007) would be a phonological change instead of a phonetic change. Specifically, Evans and Iverson’s (2007) Northern-English students would need to split their *cud–could* vowels: in the North, both of these words have /ʊ/, but in SSBE, *cud* takes the vowel /ʌ/ instead, which does not exist in the Northern phoneme inventory. The successful adoption of this difference would constitute a phonological change, namely a phoneme split. However, Evans and Iverson (2007) did not find direct evidence that their participants managed to do this in either production or perception: while some individuals changed their production targets for these vowels more into the SSBE direction, and their perception correlated with their production changes, they did so for both vowels simultaneously.

The studies mentioned in the previous two paragraphs differ in at least two important ways. The (successful) vowel changes in Evans and Iverson (2007) are contact-induced phonetic changes, while SSBE /uː/-fronting is a system-internal phonological change and, as a result, also a phonetic change. The obvious third alternative for study—and the subject of the present paper—is a contact-driven phonological change. As the interactions between the many factors mentioned in this introduction—perception versus production, system-internal versus contact-driven, infant versus adult, phonological versus phonetic change—are still very much the topic of on-going research, filling this gap makes a small contribution to the larger puzzle of sound change in general. A currently on-going sound change in Dutch offers a unique opportunity to study precisely this type of change.

### 1.4 The present study

The present study investigates the role of individual variation in sound change, using an on-going vowel shift in Dutch which has led to notable sociolinguistic variation. Dutch is spoken both in the Netherlands and in Flanders, the northern part of Belgium. In the Netherlands, a sound change is currently on-going whereby the tense mid vowels /eː,øː,oː/ are becoming diphthongs [ei,øy,ou] (van der Harst, 2011; van der Harst et al., 2014; Van de Velde, 1996; Zwaardemaker & Eijkman, 1924), except when followed by coda /l/ (realized as [ɫ], with optional vocalization in the Netherlands; van Reenen & Jongkind, 2000) or another approximant consonant (/r,υ,j/) in specific phonological configurations (Berns & Jacobs, 2012; Botma et al., 2012; Voeten, 2015). In addition, the original diphthongs /εi,œy,ɔu/ have begun to diphthongize more strongly in the Netherlands (Blankestein, 1994; Gerritsen & Jansen, 1980; Gussenhoven & Broeders, 1976; van Heuven et al., 2005; Jacobi, 2009; Mees & Collins, 1983; Stroop, 1992, 1998; Van de Velde, 1996; Voortman, 1994) except before coda /l/, while they diphthongize more weakly in Flanders (Van de Velde, 1996; Verhoeven, 2005). In both varieties, vowels are categorically realized as monophthongs before coda /l/ (Goossens et al., 1998: maps 63/66; Voeten, 2015). Thus, the Netherlandic vowel system has six diphthongs which have corresponding monophthongal allophones before coda /l/, whereas the Flemish system has three monophthongs and three very light diphthongs, which are also monophthongized before coda /l/. (For extensive discussions of Netherlandic–Flemish vowel-system differences, see Adank et al., 2004 and Van de Velde et al., 2011, in addition to the aforementioned references.)

The paper focuses its investigation on the adoption of the Netherlandic diphthongization patterns in *sociolinguistic migrants*—individuals born in Flanders who moved to the Netherlands post-adolescence and have lived there for a significant amount of time. It is investigated whether these sociolinguistic migrants adopt the Netherlandic realizations as well as their phonological conditioning related to coda /l/. Thus, the present paper fits into the second-dialect acquisition literature (which is also the perspective of many of the studies discussed at the end of section 1.2), and is indirectly related to the second-language acquisition literature (e.g., Flege, 1987; Flege & Wayland, 2019) as well. A comprehensive treatment of these perspectives is beyond the scope of this paper; the reader is referred to the aforementioned works instead. The present paper makes *use* of the situation of sociolinguistic migrants to serve as a model of the acquisition of sound change. This is possible because the synchronic sociolinguistic differences happen to coincide with the diachronic changes in the Netherlandic vowel system, such that the Flemish vowel system can be seen as a model for the Netherlandic system before these changes had taken place. This makes it possible to use laboratory experiments comparing sociolinguistic migrants to two suitable control groups to investigate how such synchronic differences are adopted by individuals on their way to becoming diachronic changes. The hypothesis is that the sociolinguistic migrants are not homogeneous; instead, some participants in this group will have become more Netherlandic-like, whereas others will have remained more Flemish-like. This hypothesis is assessed separately for production and perception, using two laboratory experiments which include suitable control groups. This makes it possible to investigate with precision whether and which of these participants have adopted the on-going sound changes in production and in perception. The link between production and perception is also examined.

Concerning production, we know that Flemish-Dutch speakers do not diphthongize their tense mid vowels /eː,øː,oː/ and only weakly diphthongize their “true” diphthongs /εi,œy,ɑu/. In contrast, Netherlandic-Dutch speakers use diphthongal realizations for all six of these vowels, especially strongly so for the diphthongs /εi,œy,ɑu/. However, all of this diphthongization is blocked categorically before a following coda /l/. Hence, predictions for the Flemish-Dutch speakers are monophthongal realizations of /eː,øː,oː/ and weakly diphthongal realizations of /εi,œy,ɑu/, whereas predictions for the Netherlandic-Dutch speakers are diphthongal realizations of /eː,øː,oː/ and strongly diphthongal realizations of /εi,œy,ɑu/, but only when there is no following coda /l/. If there is a coda /l/, the Netherlandic group is expected to realize a monophthong, with possible weak diphthongization due to coarticulation, leveling the hypothesized group differences. The migrant group is hypothesized to be heterogeneous: given the background sketched in section 1.1, it is expected that some participants will have been relatively successful at adopting the Netherlandic-Dutch sound changes, whereas others will have done so only minimally. On average, the group should then be in between the Netherlandic and Flemish groups, though with significant intra-group variation.

Turning to perception, the linguistic facts remain the same, but their implications are different. Previous research has shown that listeners of Dutch are sensitive to the specific trajectory of diphthongal vowels in perception (Peeters, 1991). Because Flemish speakers of Dutch do not produce diphthongization for the vowels /eː,øː,oː/, it is expected that their category boundary between the tense mid vowels and the diphthongs will be relatively close towards the monophthongal realizations. In concrete terms, even a little diphthongization will be a cue for a Flemish-Dutch speaker to perceive a vowel as /εi,œy,ɑu/. Alternatively, a reviewer suggests that it is also possible that the Flemish group does not perceive such light diphthongization at all, precisely because they do not have it in their repertoire. For a Netherlandic-Dutch speaker the same weak diphthongization should be a perfectly regular cue for a percept of /eː,øː,oː/, but only in absence of a following coda /l/. In this situation, it is expected that the Netherlandic group has their category boundary further towards the diphthongal realizations than the Flemish group; the Netherlandic group should require more diphthongization to be present in an ambiguous signal to perceive it as /εi,œy,ɑu/. This effect is expected to be quite strong, because in addition to the diphthong–monophthong differences between the two varieties (Adank et al., 2004; van der Harst, 2011; van der Harst et al., 2014; Van de Velde, 1996; Van de Velde et al., 2011), the Netherlandic realizations of /εi,œy/ have significantly lower onsets and are more similar to [ai,ɒy] (van Heuven et al., 2005; Jacobi, 2009; Stroop, 1998). If the vowel is followed by /l/, these between-groups differences are hypothesized to vanish: here, neither group has grounds to expect diphthongization for reasons beyond effects of phonetic implementation. As for production, the migrant group is expected to be in between the two control groups at the group level, but is expected to show significant individual variation, correlated with the individual variation found in the production experiment.

Section 2 investigates the hypotheses for the production part, using a simple word-list-reading task eliciting a representative subset of the relevant vowels both before /l/ and before nonapproximant consonants. Due attention is paid not just to variation at the group level, but also to individual differences. Individual differences are investigated by analyzing the predicted random effects of a mixed-effects model, which has only recently gained traction in sociophonetics (e.g., Drager & Hay, 2012; Tamminga, to appear). The analysis controls for one major factor, namely lexical frequency, which is known to play a role both in sound change (Bybee, 2002) and in the adoption of sociolinguistic differences (Nycz, 2013).

The perception hypotheses are investigated in section 3. Participants’ category boundaries between monophthong and diphthong phonemes are elicited on the basis of a novel experiment based on participants’ rhyme decisions to nonsense words. In general, rhyme-decision tasks have been used successfully in the related field of second-dialect acquisition (Nycz, 2011) and have the advantage of being less direct than the more traditional task of phoneme decision (as had been used in, e.g., Pinget, 2015). Compared to phoneme decision, rhyme decision is less obvious about the nature of the experiment and is more linguistic rather than meta-linguistic in nature. Both points serve to reduce the likelihood of participants resorting to explicit cognitive strategies that do not reflect their everyday linguistic processing, and of them letting any overt or covert linguistic norms influence their responses. This is important especially for the migrant participants, who may have become subliminally or supraliminally aware of the relevant accent differences. The rhyme-decision task tests three phonetic continua: [eː~εi] (testing diphthongization), [oː~ɑu] (testing diphthongization plus marked lowering of the nucleus), and a control contrast [ε~εi] (testing diphthongization and duration). Rhyme decisions between an ambiguous auditory prime and an orthographic target are used to obtain covert phoneme decisions: at what point along the continuum do participants stop perceiving /eː,oː/ and start perceiving /εi,ɑu/? The condition using [ε~εi] is included as a control: these vowels are like the experimental items in that /ε/ is a monophthong and /εi/ is a diphthong, but the boundary between these otherwise unrelated categories is unaffected by the on-going sound changes studied in this paper.

Section 4 investigates the individual-level correlations between production and perception, which were suggested to exist in the formulated hypotheses. This is done by calculating correlation coefficients between the participants’ coefficients from the individual-level analyses of the production and perception tasks. The findings are brought together in the general discussion in section 5. Finally, section 6 concludes the paper with possible avenues for further study.

## 2 Experiment 1: Production

### 2.1 Method

#### 2.1.1 Participants

Three groups of participants were recruited. The first group consisted of 45 Dutch students at Leiden University, the Netherlands. These students were native speakers of Netherlandic Standard Dutch and were born and raised in the Randstad,^2^ the area of the Netherlands where the prestigious variety of Dutch is spoken that forms the basis for Netherlandic Standard Dutch (Grondelaers & van Hout, 2011). The second group consisted of 45 Belgian students at Ghent University, Belgium. These students were all native speakers of Belgian Standard Dutch and were born and raised in Flanders. The third group of participants consisted of 18 Belgians who were native speakers of Belgian Standard Dutch and had been living in the Randstad area of the Netherlands for a long time (mean = 18.71 years, SD = 11.18 years). Two participants in the Ghent group were excluded due to technical failures, resulting in a total of 106 participants used.

**Figure 1.**
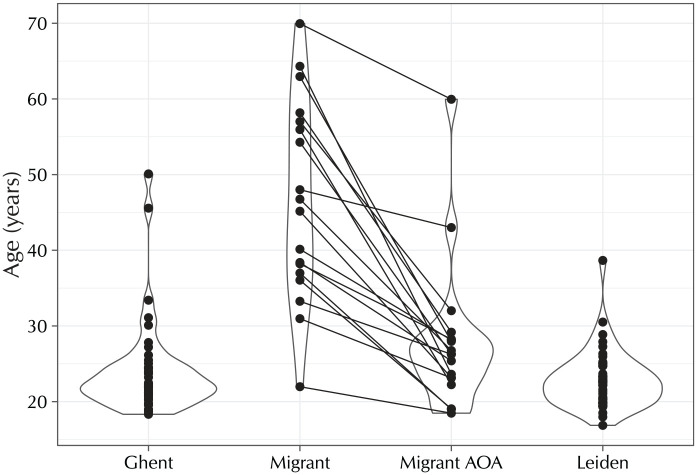
Dot and violin plots of the ages of the participants in the Ghent (*n* = 43, 1 missing age value), migrant (*n* = 18), and Leiden (*n* = 45, 2 missing age values) groups. For the migrant group, the chronological ages have been linked to the participants’ ages of arrival (AOA) in the Netherlands.

Table 1 details participants’ reported regional backgrounds, split out by province. In addition to speaking the standard language (either Netherlandic Dutch or Flemish Dutch), seven of the Ghent participants and seven of the migrant participants reported being proficient in their local Flemish dialects. None of the Leiden participants reported dialect competence. Figure 1 shows the variation in the participants’ ages and, for the migrant participants, their lengths of stay in the Netherlands. The migrant participants’ ages are heterogeneous, but their ages of arrival are not: with two exceptions, participants were in their mid-twenties (range: 18–32 years old) when they migrated to the Netherlands. Of the two exceptional participants, one had just turned 43 when they moved countries, and the other was a few days short of their 60th birthday upon migration.

**Table 1.** Regional backgrounds of the participants, defined as the province in which they attended high school. Gelderland and –Zeeland are Netherlandic provinces, while the others are Flemish.

**Region**	**Group**		
	Ghent	Migrant	Leiden
Gelderland	0	0	2
Netherlandic Limburg	0	0	1
North Holland	0	0	6
South Holland	0	0	28
Utrecht	0	0	5
Zeeland	0	0	2
Antwerp	5	1	0
Brussels	0	1	0
East and West Flanders	0	1	0
East Flanders	23	4	0
Flemish Brabant	6	4	0
Flemish Limburg	0	2	0
West Flanders	14	5	0
(missing)	0	0	1

The experiments followed the Ethics Code for linguistic research in the faculty of Humanities at Leiden University, which approved its implementation. All subjects gave written informed consent in accordance with the Declaration of Helsinki.

#### 2.1.2 Stimuli

**Figure 2.**
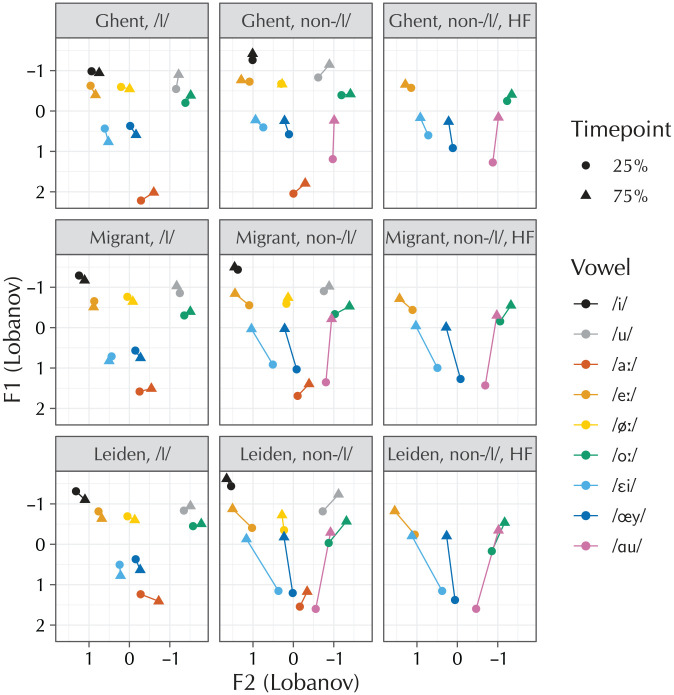
Vowel diagrams of the data collected in the study (106 participants, 361 items). The figure is divided into nine panels, to account for the 3 groups × 3 types of following segment in the design. The vowels of experimental interest are /eː,oː,εi,œy/; the other vowels are included for context.

Words were selected from the combined CELEX (Baayen et al., 1995) and SUBT-LEX (Keuleers et al., 2010) corpora^3^ to elicit (possible) diphthongs followed by either coda /l/ or a nonapproximant consonant. Words were selected such that the critical vowel always received primary stress. Words sharing the same lemma were avoided. For the words where the critical vowel was followed by a nonapproximant consonant, a distinction was made between low-frequency and high-frequency (henceforth: HF) words, defined by the set of words falling in the first and third quartiles, respectively, of the log10 word frequency. This distinction was not made for the coda-/l/ words, due to there not being enough high-frequency V+/l/ words in the corpus. The vowels /øː/ and /ɑu/ were included in the study but excluded from the data analysis, because the former vowel was not frequent enough to fill the design cells with the full 20/40 tokens and the latter cannot be followed by coda /l/ due to a lexical gap (which, as also mentioned by van Reenen and Jongkind [2000] and Peeters [1991], is due to a historical process of /l/-vocalization and diphthongization). Table 2 summarizes the stimuli design. In addition to the vowels mentioned there, the practice trials of the experiment were used to elicit the point vowels /i,u,aː/, both preceding /l/ (5 tokens per vowel) and preceding a nonapproximant consonant (5 tokens per vowel), but without regard for frequency. These were also excluded from the analysis, and only serve to provide anchor points for the vowel-space plots in Figure 2. The full list of items is available in Appendix A.

**Table 2.** Number of words in each cell of the stimuli design of the production experiment, with the column “Analyzed” reflecting which of these were included in the data analysis. In addition to the vowels mentioned here, the practice part of the experiment elicited 30 tokens (5 per combination) of / i,u,aː/ before coda /l/ and before non-/l/.

**Vowel**	**Analyzed**	**Following segment + frequency**		
		coda /l/	non-app. + LF	non-app. + HF
/eː/	Yes	20	20	20
/oː/	Yes	20	20	20
/εi/	Yes	20	20	20
/œy/	Yes	20	20	20
/øː/	No	11	20	0
/ɑu/	No	0	20	20

#### 2.1.3 Procedure

For the Leiden and migrant participants (who were tested in Leiden), the experiment took place in a dimly lit sound-attenuating booth, where participants were seated in front of a computer screen and a studio-quality microphone. For the Ghent participants, the experiment took place in a quiet room, where participants were seated in front of a laptop and wore a studio-quality headset. The participants performed a word-list-reading task, presented using E-Prime (Psychology Software Tools, 2012) running on Windows 7 (both setups). The words selected for the experiment were presented on the screen one by one (in pseudorandomized order), and participants were instructed to read these words aloud into the microphone. Each trial had a fixed duration of 2s and was followed by a fixation cross presented for 500ms. A total of 2.5s was thus available for speaking (the presentation of the fixation cross did not terminate the recording).

#### 2.1.4 Data analysis

The acquired single-word recordings were forcedly aligned to their CELEX reference transcriptions using HTK (Young et al., 2002). Using Praat (Boersma & Weenink, 2016), the F1s of the vowels of interest were extracted at 25% and 75% realization of the vowel (Burg algorithm, time step 10ms, 5 formants, cut-off point 5000Hz for men and 5500Hz for women, window length 25ms, pre-emphasis from 50Hz). The choice to use 25% and 75% points is based on findings by van der Harst (2011) that these are the first and last time points, respectively, that are not too strongly influenced by coarticulation. Following van der Harst (2011, p. 82), outliers were identified for male and female speakers separately and for the two measurement points separately, with outliers defined as F1 values whose absolute values exceeded 1.5 times the interquartile range within the relevant combination of gender and timepoint. These outliers’ formant values were removed from the data, based on the argument that they constituted either formant-tracking errors or forced-aligner errors. Of the 71,054 measurements in total, 67,283 (94.7%) remained after this procedure. The data were subsequently normalized using the Lobanov method; this was found to be the best-performing method for this type of data in the comparisons by Adank et al. (2004) and van der Harst (2011).

In order to capture diphthongization in a single variable suitable for statistical modeling, the two time points per token were converted into a single difference score, to which I henceforth refer as “DF1.” This score was computed for each token by subtracting the measurement at 25% from the measurement at 75% and encodes the amount of diphthongization present in the vowel. Negative values indicate upgliding diphthongization, while values of zero or slightly above it indicate lack of upgliding diphthongization. The resulting data contained 32,861 difference scores, which amounts to 92.5% ^4^ of the original data. Finally, only the vowels /eː,øː,εi,œy/ were selected from these data, yielding a final dataset of 23,393 difference scores.

The F1 difference scores were analyzed using a mixed-effects model in two ways. In both approaches, the dependent variable was DF1. Fixed effects were included for “Vowel” (levels/eː,øː,oː,εi,œy,ɑu/; sum-coded such that /εi/ = −1 and the other vowels = 1; this coding scheme makes the estimated contrasts relative to the grand mean of all vowels), “Following segment” (levels /l/ or non-/l/, treatment-coded such that non-/l/ = 1 and /l/ = 0), frequency (deviation-coded such that High Frequency = 0.5 and other = −0.5; this coding scheme uses the mean as the reference, but estimates the difference between the two frequency types, rather than their difference from the mean), and all appropriate interactions. In the analysis in section 2.2.1, a factor “Group” has also been included, coded using Helmert coding such that the first contrast compares the Ghent group and the migrant group, and the second contrast compares the Leiden group to the average of the other two. A maximal model structure was formed including all interactions and full random slopes by participants and by words, but excluding correlations between random slopes. The model was fitted to scaled-t errors, using function bam from R package mgcv (Wood 2017). From this maximal model, terms that did not achieve omnibus significance^5^ were removed using backward elimination (see Matuschek et al., 2017 for justification) to arrive at a parsimonious final model (per Bates et al., 2015).

Based on the studies reported in section 1.1, it can be considered questionable to lump the 18 migrant participants together into a single explicit group. For this reason, section 2.2.2 presents an alternative analysis, where the interest was not in group patterns, but in individual differences. The objective of this second analysis was to find homogeneous groups in the set of participants, in order to identify which participants are more Ghent-like and which participants are more Leiden-like. This analysis did not include the factor “Group” and fitted the full model directly, without performing stepwise elimination. For each of the by-participants random-effect terms estimated by the model, the predicted b values were extracted. These values are the individual participants’ coefficients for the estimated random slopes. Function Mclust from R package mclust (Scrucca et al., 2016) was used to perform a cluster analysis on these coefficients, based on a one-dimensional variable-variance cluster model. This analysis provides a quantitative measure of the degree to which individual participants are more Ghent-like or more Leiden-like.

The data and R code for the analyses are available at https://figshare.com/s/731e0a32480e876530e0 as the files production.csv and production.R, respectively.

### 2.2 Results

Figure 2 shows vowel-space diagrams of the collected data, without any prior analysis. The figure shows the four vowels of critical interest, plus the point vowels /i,u,aː/ and the excluded diphthongs /øː,ɑu/ for context.

#### 2.2.1 Analysis by groups

Table 3 shows the results of the analysis in which the three groups of participants were categorized into their respective three groups a priori. The results show that a following non-/l/ induces significant upgliding diphthongization (β̂ = −0.79, SE = 0.04, t = −22.12, p< .001), but there are large and highly significant differences between the groups in this respect. The significant “Following segment = non-/l/ × Migrant–Ghent” interaction shows that migrants on average produce standard deviations more diphthongization (SE = 0.03, t = −7.14, p < .001) than the Ghent group does. There is also a significant interaction of “Following segment = non-/l/ × Leiden–Others,” indicating that the Leiden participants produce even more diphthongization than the other two groups do: on average, they diphthongize an additional −0.20 standard deviations more (SE = 0.02, t= −12.13, p < .001) than the Ghent and migrant groups. There are significant per-vowel adjustments to the regression coefficients discussed thus far, but in all three groups these are small enough that they do not rise above the crucial effect of a following non-/l/ consonant.

**Table 3.** Group-level results for the production data (106 participants, 235 items). Critical factors are “Following segment” and its interaction with the group predictors. The key observations are that the average participant produces significantly more diphthongization before non-/l/ than before coda /l/, and that this additionally varies between the three groups. The migrant group produces significantly more diphthongization in this context than the Ghent group, and the Leiden group produces even more diphthongization in this context than the average of the other two groups.

Factor	Estimate (SE)	*t*	*p*	Sig.
Intercept	0.20 (0.03)	6.86	< .001	***
Vowel = /eː/	0.02 (0.04)	0.45	.65	
Vowel = /oː/	−0.24 (0.05)	−5.38	< .001	***
Vowel = /œy/	0.11 (0.04)	2.43	.02	*
Following segment = non–/l/	−0.79 (0.04)	−22.12	< .001	***
Group = Migrant–Ghent	−0.06 (0.02)	−2.92	< .01	**
Group = Leiden–Others	0.01 (0.01)	0.46	.64	
Following segment = non–/l/ × Frequency = HF	−0.05 (0.02)	−2.58	< .01	**
Vowel = /eː/ × non-/l/	0.31 (0.06)	5.59	< .001	***
Vowel = /oː/ × non-/l/	0.56 (0.06)	9.91	< .001	***
Vowel = /œy/ × non-/l/	−0.53 (0.05)	−9.68	< .001	***
Following segment = non-/l/ × Migrant–Ghent	−0.19 (0.03)	−7.14	< .001	***
Following segment = non-/l/ × Leiden–Others	−0.20 (0.02)	−12.13	< .001	***
Vowel = /eː/ × non-/l/ × Migrant–Ghent	0.12 (0.03)	4.45	< .001	***
Vowel = /oː/ × non-/l/ × Migrant–Ghent	0.11 (0.03)	3.91	< .001	***
Vowel = /œy/ × non-/l/ × Migrant–Ghent	−0.15 (0.02)	−6.20	< .001	***
Vowel = × non-/l/ × Leiden–Others	0.07 (0.02)	4.21	< .001	***
Vowel = × non-/l/ × Leiden–Others	0.03 (0.02)	1.81	.07	
Vowel = /œy/ × non-/l/ × Leiden–Others	−0.04 (0.02)	−2.85	< .01	**

#### 2.2.2 Analysis by participants

**Figure 3.**
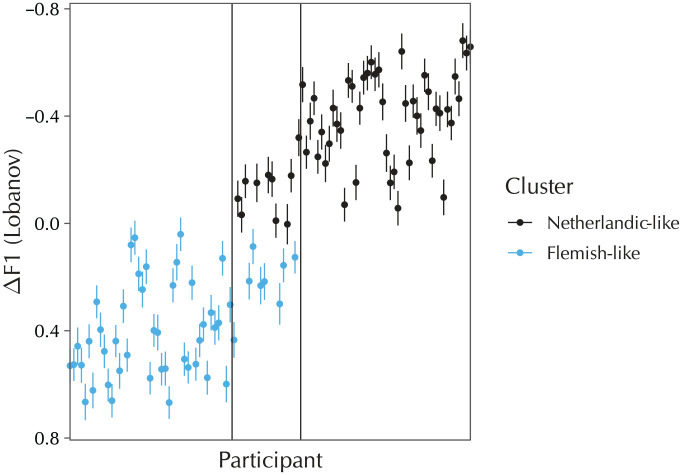
BLUPs for the “Following segment = non-/l/” term in the by-individuals (*n* = 106) model. Each dot is a participant’s individual random-effect coefficient; lines indicate the standard errors. The left pane shows the participants from the Ghent group, the middle pane shows the participants from the migrant group, and the right pane shows the participants from the Leiden group.

Figure 3 shows a plot of the individual participants’ random-effect coefficients (henceforth BLUPs, for “best linear unbiased predictors”) for the “Following segment = non-/l/” term; this is the single predictor of critical interest. The cluster analysis found 2 clusters for this factor, which have a clear interpretation: the cluster analysis managed to completely recover the Leiden and Ghent groups, despite not having been provided with any a priori group information. This was not the case for the other random-effect terms, where either only one cluster was found, or where two clusters were found but these failed to coincide with either of the group boundaries. Since these other terms were not of theoretical interest anyway, they have been relegated to Appendix B.

The BLUPs in Figure 3 show that one set of participants diphthongizes significantly more than average before a nonapproximant consonant, and one set of participants diphthongizes significantly less than average in this environment. All of the Leiden participants are in the former cluster, and all of the Ghent participants are in the latter cluster. Concerning the migrant group, 10 of these participants diphthongize to such an extent that they are classified with the Leiden group, whereas the other eight are still classified with the Ghent group. An anonymous reviewer asks whether the two migrant participants who had arrived to the Netherlands relatively late were among those clustered with the Ghent group. This was indeed the case.

### 2.3 Discussion

The two largest effects in the group-level analysis are the effect for “Following segment = non-/l/” and its interaction with “Group = Leiden–Others.” The former shows that the main difference is between all vowels before non-/l/ versus before /l/. The latter shows, wholly in line with the hypothesis, that the Leiden participants diphthongize significantly more than the Ghent participants. As a group, the migrant participants are in between; they are significantly different from the Ghent participants, but do not diphthongize as much as the Leiden participants (their effect was approximately one-third the size of that of the Leiden group).

The results at the individual level confirm and extend these findings. The cluster analysis shows that nearly all Leiden participants produce significantly more diphthongization than nearly all Ghent participants. The migrant participants are in between; some diphthongize to such extent that they are classified with the Leiden participants, some do not and are classified with the Ghent participants. Thus, the cluster analysis makes it possible to identify precisely which individuals make positive or negative contributions to the overall group-level effect. The critical difference in diphthongization was captured by the BLUPs for the “Following segment = non-/l/” random slope, which is the grand mean of all five possibly diphthongizing vowels. This shows that these differences in diphthongization are across the board, and are not specific to one vowel or one subset of vowels.

The results suggest a role for age of arrival in determining the migrant participants’ degrees of sound-change adoption, insofar as the two participants who arrived well past their twenties were clustered with the Flemish group, whereas the migrants who were classified with the Netherlandic group were all in their mid-twenties when they arrived in the Netherlands. The data prohibit a formal test of this observation, both because of the small sample size and because it is confounded with participants’ lengths of exposure. However, on purely theoretical grounds it seems likely that individuals with younger AOAs would indeed more readily adopt accent differences such as those discussed here. Such participants are likely more cognitively flexible, and may also have a greater desire to fit into their peer group. They may hence be both able and willing to adopt their peers’ accents. The results by Evans and Iverson (2007) corroborate this view, but the results by Nycz (2013)—who found no effect of AOA, despite having tested a relatively comparable participant group^6^—again muddle the picture. Further investigation of a link between age of arrival, length of exposure, and cognitive and social factors influencing the adoption of accent differences and sound change is left to future research.

## 3 Experiment 2: Rhyme decision

### 3.1 Method

#### 3.1.1 Participants

The participants were the same as in Experiment 1, which was performed on the same occasion. Half of the participants had participated in Experiment 1 prior to taking Experiment 2, and for the other half the experiments were performed in the opposite order.

#### 3.1.2 Stimuli

The experiment used vowel pairs of [eː~εi], [oː~ɑu], and [ε~εi]. As detailed in section 1.4, the former two are of experimental interest, whereas the latter served as a control condition. For the orthographic targets, 2×192 pseudowords were generated according to a template of [C1_(ɫ).{t,d}ə(ɹ)]; one pseudoword was generated for each vowel in each pair. The presence/absence of the parenthesized /l/ and /r/ and the choice between /t/ and /d/ were perfectly balanced, leading to 16 items per cell for a total of 384 targets, half of which (viz. those with the word-final [ɹ]) look like plausible Dutch nouns and half of which (viz. those without the word-final [ɹ]) look like plausible Dutch inflected verbs. Pseudoword pairs were generated to maximize (a) syllable frequencies and (b) transition probabilities, for both words in each pair together, so as to maximize the naturalness of the words included in the experiment. Real words (defined as words occurring in CELEX; Baayen et al., 1995) were excluded to prevent the possible confounding of the experiment by this extra factor.

**Figure 4.**
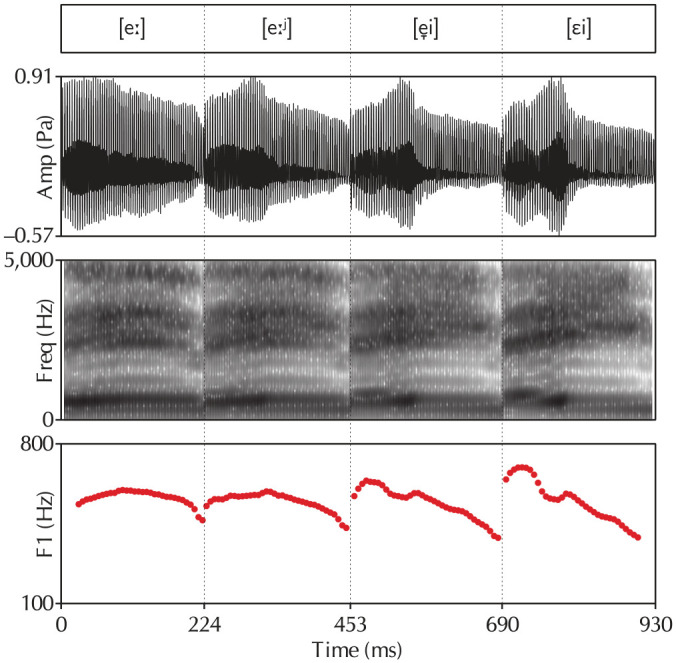
Example waveforms, spectrograms, and F1 trajectories for the [eː~εi] contrast. The four tokens shown in this figure correspond to the stimuli containing 20%, 40%, 60%, and 80% morphing, respectively. Note the increase in the diphthong’s slope and the lowering of its nucleus over the four figures.

An equal number of auditory prime words were generated in exactly the same way as the orthographic targets. All generated prime words were read aloud in a carrier sentence by a female native speaker of Netherlandic Standard Dutch from the Randstad area of the Netherlands, who read the stimuli using her regular accent. For each vowel, two tokens were selected, one followed by coda /l/ and one followed by a syllable boundary. Using Tandem-STRAIGHT (Kawahara et al., 2008), a continuum of four intermediate steps (20%–40%–60%–80% morphing from the monophthong to the diphthong realization) was generated for each vowel pair using holistic morphing between the two selected vowels. Figure 4 shows an example of the resulting waveforms, spectrograms, and F1 trajectories (the critical difference between monophthongal and diphthongal realizations) for the [eː~εi] contrast. These manipulated vowels were spliced into the original prime words. Any F0 discontinuities were smoothed out using the PSOLA algorithm in Praat (Boersma & Weenink, 2016).

The prepared prime pseudowords were paired to the target pseudowords, such that the prime and target would rhyme if and only if participants perceived the same vowel in the prime word as they read in the target word. As an example, the orthographic target pair <nebe,nijbe> (/neːbə,nεibə/) was paired with a set of auditory primes which can be rendered approximately as [beːbə,beː^j^bə,beibə,bεibə], which respectively correspond to the 20%–40%–60%–80% steps. The resulting stimuli were randomized across four lists, which were assigned to participants in fixed order (participants 1, 5, and 9 received list A, participants 2, 6, and 10 received list B, etc.). The pairing of target and prime words was yolked across these lists, such that between all of the participants, all combinations of target word and morphing step in the corresponding prime word were represented (list A paired <nebe,nijbe> with [beːbə] and <kede,kijde> with [veː^j^də], list B paired the same two targets with primes [beː^j^bə] and [veidə], etc.). This ensured that all participants would be presented with all steps of the continua and with all words in the experiment, without repeating individual pseudowords with a different morphing step (which would be another possible confounding factor). The full list of prime–target pairs is available in Appendix C.

#### 3.1.3 Procedure

Participants were seated in the same booth as in Experiment 1. Instructions about the procedure of the experiment were presented on the computer screen. Participants could start the experiment whenever they were ready by pressing one of the five buttons on the Serial Response box that was in front of them. For each trial in the experiment, the prime word was played while the target word was displayed on the screen. Participants had to indicate, by pressing either the leftmost or the rightmost button on the response box, whether the prime and target word rhymed or not. For half of the participants, the leftmost button indicated “yes” and the rightmost button indicated “no”; for the other half of the participants, this was swapped to counterbalance for any possible left–right response bias to the stimuli. All experimental items were presented twice: once for each of the two orthographic targets assigned to the auditory prime for each participant. After completion of a trial, a fixation cross appeared for 500ms, after which the next trial was presented. There were three breaks spaced evenly throughout the experiment. Before the real experiment began, participants were presented with a few practice trials. These consisted of a total of 12 prime–target combinations, generated and administered according to exactly the same procedure as the main experiment.

While the general approach of rhyme decision has been used before (Nycz, 2011), the paradigm proposed in the present study, which uses nonsense words, is novel. Therefore, the experiment was subjected to extensive piloting, on two occasions: once with a group of colleagues in Leiden University’s linguistics department, to solicit comments on anything from the general principles behind the experiment to subjective experiences of individual stimuli, and once with a group of Netherlandic-Dutch speakers and a control group of Flemish sociolinguistic migrants (different from the group tested in the present paper) to validate that the expected effects were indeed borne out. The lessons learned from the first pilot were used to improve the second pilot, which validated that this experimental paradigm could indeed capture participants’ perceptual categories in sufficient detail. These results, reported in more detail in Voeten (2020: chapter 3), showed the expected, approximately linear, increase from a very small towards a very large probability of reporting a diphthong percept as the morphing step increased.

#### 3.1.4 Data analysis

To directly test the effect of morphing step on participants’ vowel perceptions, participants’ yes/no responses to the prime–target combinations were recoded into vowel percepts associated with each auditory prime. Responses with reaction times < 100ms or > 5000ms were excluded from the dataset. The remaining data were analyzed using a similar approach to the one used in section 2.1.4. Separate^7^ mixed-effects logistic-regression models were fitted for the three conditions [eː~εi], [oː~ɑu], and [ε~εi]. The dependent variable was “Phoneme decision,” which coded whether the participant’s judgment was consistent with the monophthongal phoneme (/eː,oː,ε/, coded as 0) or the diphthongal phoneme (/εi,ɑu,εi/, coded as 1). Fixed effects were added for “Step” (coded for linear, quadratic, and cubic trends using orthogonal polynomials) and “Following segment” (deviation-coded such that non-/l/ = −0.5 and /l/ = 0.5; this coding scheme tests for the difference between the two stimuli while using their average as the reference). Random intercepts and slopes for all predictors by participants were included, as was a random intercept for “Item” (the prime-target pair presented in each trial). As in section 2.1.4, models were run both with and without an explicit factor “Group.” When included, the factor “Group” was coded in the same way as in section 2.2.1, with all fixed-effect interactions and a random slope by items. For the by-groups model, function buildglmmTMB from R package buildmer (Voeten, 2019) was used to identify the maximal random-effect structure that still converged, and terms were eliminated using backward stepwise elimination based on the likelihood-ratio test. The data and R code for the analyses are available at https://figshare.com/s/731e0a32480e876530e0 as the files rhyme.csv and rhyme.R, respectively.

### 3.2 Results

Cluster analyses on the by-participants model revealed no robust groupings: all analyses yielded only one cluster. For this reason, only the results from the three by-groups models are reported here. These results are shown in Table 4. This table only shows results that achieved significance according to a Bonferroni-corrected αof .017; significance stars have been corrected to reflect two-tailed p-values of .017 (*), .0033 (**), and .00033 (***). Appendix D presents also the results that did not reach significance.

**Table 4.** Results of the rhyme-decision task (106 participants, 1,536 items). Only significant results are shown; the reader is referred to Appendix C for the full result set. The key results are (1) the significant linear trends of participants indicating more diphthong percepts at later morphing steps; (2) participants becoming more likely to give diphthong responses to a following coda /l/, demonstrating perceptual compensation in the non-/l/ words; (3) significant between-groups differences in the effect of morphing step in the [eː~εi] and [ε~εi] models.

Factor	Estimate (SE)	Odds ratio	*z*	*p*	Sig.
**Model = [eː~εi]**					
Intercept	−1.46 (0.10)	1 : 4.30	−14.49	< .001	***
Step (Linear)	1.24 (0.13)	3.47 : 1	9.87	< .001	***
Step (Quadratic)	0.33 (0.08)	1.39 : 1	4.22	< .001	***
Following segment = /l/	0.83 (0.11)	2.29 : 1	7.59	< .001	***
Group = Migrant–Ghent	−0.51 (0.13)	1 : 1.66	−3.95	< .001	***
Step (Linear) × /l/	1.68 (0.21)	5.37 : 1	8.14	< .001	***
Step (Linear) × Migrant–Ghent	0.63 (0.15)	1.87 : 1	4.06	< .001	***
**Model = [oː~ɑu]**					
Intercept	0.20 (0.07)	1.22 : 1	2.76	.01	*
Step (Linear)	1.73 (0.15)	5.65 : 1	11.48	< .001	***
Step (Quadratic)	0.45 (0.11)	1.57 : 1	4.17	< .001	***
Following segment = /l/	0.95 (0.12)	2.59 : 1	7.83	< .001	***
Step (Linear) × /l/	−0.66 (0.25)	1 : 1.93	−2.67	.01	*
Step (Quadratic) × /l/	−0.74 (0.22)	1 : 2.09	−3.32	< .001	**
**Model = [ε~εi]**					
Intercept	−0.94 (0.07)	1 : 2.55	−13.12	< .001	***
Step (Linear)	2.49 (0.15)	12.06 : 1	16.44	< .001	***
Step (Quadratic)	0.27 (0.10)	1.31 : 1	2.59	.01	*
Step (Cubic)	−0.74 (0.11)	1 : 2.09	−6.99	< .001	***
Following segment = /l/	0.96 (0.12)	2.60 : 1	7.90	< .001	***
Group = Migrant–Ghent	−0.21 (0.08)	1 : 1.23	−2.64	.01	*
Group = Leiden–Others	0.09 (0.04)	1.10 : 1	2.50	.01	*
Step (Linear) × /l/	−1.38 (0.24)	1 : 3.98	−5.84	< .001	***
Step (Cubic) × /l/	0.87 (0.21)	2.38 : 1	4.13	< .001	***
Step (Linear) × Migrant–Ghent	0.43 (0.17)	1.53 : 1	2.52	.01	*
Step (Linear) × Leiden–Others	−0.23 (0.08)	1 : 1.26	−2.91	< .01	**
Step (Linear) × /l/ × Leiden–Others	−0.31 (0.11)	1 : 1.36	−2.95	< .01	**

**Figure 5.**
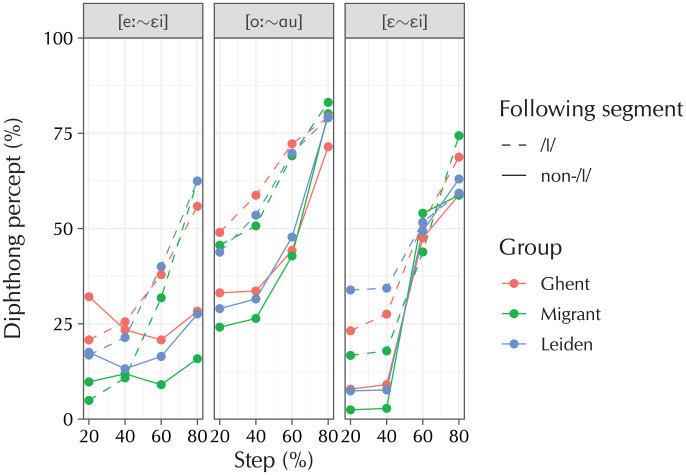
Averaged raw data from the rhyme-decision task (106 participants, 1,536 items). The general trends are that (1) participants become more likely to indicate a diphthong percept at later morphing steps; (2) this effect is larger before coda /l/ than before non-/l/, indicating that participants are perceptually compensating in the latter but not the former condition; (3) there are differences between the groups both at the baseline and as a function of the morphing step.

To aid interpretation of the model coefficients, Figure 5 plots the raw data on which these models have been based. The three panels of this figure correspond to the three fitted models, which will now be discussed in turn.

The model for the [eː~εi] condition reveals significant linear and quadratic effects of “Step” ($\hat{\beta }$β̂ = 1.24, SE = 0.13, OR = 3.47: 1, z = 9.87, p < .001 ; β̂ = 0.33, SE = 0.08, OR = 1.39: 1, z = 4.22, p < .001), indicating a steeper-than-linear trend of obtaining a diphthong percept at later morphing steps. A following /l/ increased the odds of a participant choosing the diphthongal target, both at the baseline (β̂ = 0.83, SE = 0.11, OR = 2.29: 1, z = 7.59, p < .001) and as a (linear) function of morphing step (β̂ = 1.68, SE = 0.21, OR = 5.37 :1, z = 8.14, p < .001). The Ghent group had lower baseline odds of obtaining a diphthong percept than the other two groups (β̂ = −0.51, SE = 0.13, OR = 1: 1.66, z = −3.95, p < .001), but their odds also increased more steeply as a linear function of the morphing step (β̂ = 0.63, SE = 0.15, OR = 1.87: 1, z = 4.06, p < .001). Taken together, these results paint a picture where participants become more likely to opt for the diphthong target at later morphing steps, which is exactly what was expected based on the manipulation. In the coda-/l/ condition, participants were already more likely to obtain a diphthong percept, and became even more so at the later morphing steps, doing so more rapidly than in the non-/l/ condition. The differences between the groups revolved around the difference between the Leiden and migrant groups on the one hand and the Ghent group on the other—the latter initially showed a preference for the monophthongal targets, but went for the diphthongal targets more rapidly than the other groups at the later morphing steps.

For the [oː~ɑu] model, the results show the same effect of participants becoming more likely to select the diphthong target at later morphing steps, which again developed according to a combined linear and quadratic trend (β̂ = 1.73, SE = 0.15, OR = 5.65 : 1, z = 11.48, p < .001; β̂ = 0.45, SE = 0.11, OR = 1.57 : 1, z = 4.17, p < .001). There were again significant effects of a following /l/ (β̂ = 0.95, SE = 0.12, OR = 2.59 : 1, z = 7.83, p < .001) and its interaction with “Step” both linearly and quadratically (β̂ = −0.66, SE = 0.25, OR = 1 : 1.93, z = −2.67, p = .01; β̂ = −0.74, SE = 0.22, OR = 1 : 2.09, z = −2.32, p < .001). Differences between the groups are not borne out in this model. These results show that all groups of participants again became more likely to obtain a diphthong percept at later morpheme steps. If a coda /l/ followed, they became even more likely to opt for the diphthong, but the gap between the two following segments narrowed at the later morphing steps. The most important effects are those that are not found: the hypothesized group differences do not appear to be borne out in the [oː~ɑu] condition.

**Figure 6.**
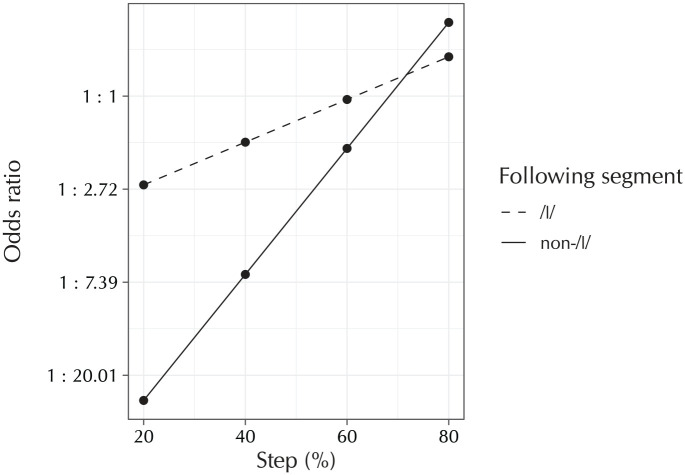
Partial-effect plot of the three-way interaction in Table 4. The plot shows the difference between the Leiden group and the others, in their interaction of “Step” (on the *x* axis) and “Following segment” (as separate lines), in the [ε~εi] condition. The *y* axis is on a logarithmic scale, as this is the scale on which the partial effects in the logistic-regression analysis are linear. Observe that the Leiden group has much lower odds of reporting a diphthong percept than the other two groups in the first three steps, but at the fourth morphing step this preference reverses and the Leiden group has slightly higher odds of reporting a diphthong percept than the two other groups. Finally, note that this effect is much more pronounced, in having a steeper linear slope leading to larger group differences in the earlier steps, in the non-/l/ case than in the /l/ case.

Finally, the [ε~εi] model is the model for the control condition, where the diphthong [εi] was morphed together with the—as far as the relevant sound changes are concerned, arbitrary—vowel [ε]. Reassuringly, the same linear and quadratic effects for “Step” are obtained (β̂ = 2.49, SE = 0.15, OR = 12.06 : 1, z = 16.44, p < .001; β̂ = 0.27, SE = 0.10, OR = 1.31 : 1, z = 2.59, p = .01). An additional cubic effect is also observed (β̂ = −0.74, SE = 0.11, OR = 1 : 2.09, z = −6.99, p < .001). These effects together create a curve that that has a sharp increase between steps 2 and 3, and much lower increases between the first two steps and between the last two steps. A following coda /l/ again increases the odds of participants choosing the diphthong target (β̂ = 0.96, SE = 0.12, OR = 2.60 : 1, z = 7.90, p < .001). As for the [oː~ɑu] model, the interaction terms of “Step” by “Following segment = /l/” show that the gap between the two following consonants closes towards the later morphing steps, with evidence for both a linear trend and a cubic trend (β̂ = −1.38, SE = 0.24, OR = 1 : 3.98, z = −5.84, p < .001; β̂ = 0.87, SE = 0.21, OR = 2.38 : 1, z = 4.13, p < .001). The cubic trend corresponds to what can be observed happening in Figure 5 at the 60% step, where the non-/l/ condition briefly overtakes the /l/ condition. There are significant differences between all three participant groups. The migrant participants are less likely to opt for the diphthong target (β̂ = −0.21, SE = 0.08, OR = 1 : 1.23, z = −2.64, p = .01), but become significantly more likely to do so at later morphing steps (β̂ = 0.43, SE = 0.17, OR = 1.53 : 1, z = 2.52, p = .01). This simply means that their decision boundary between steps 2 and 3 is steeper. The Leiden participants are in between, both at the baseline (β̂ = 0.09, SE = 0.04, OR = 1.10 : 1, z = 2.50, p = .01) and in interaction with “Step (Linear)” (β̂ = −0.23, SE = 0.08, OR = 1 : 1.26, z = −2.91, p < .001). The latter effect becomes stronger in the presence of a following /l/; because this is a three-way interaction, Figure 6 provides a visualization to ease interpretation. It can be observed in this figure that the difference between the Leiden group and the other two groups follows a steeper slope in the non-/l/ than in the /l/ condition, with the non-/l/ condition eliciting much fewer diphthong responses in the first three morphing steps. However, by the final morphing step, these effects have crossed over, such that there the non-/l/ condition elicits slightly more diphthong responses than the /l/ condition than the /l/ condition does between these participant groups. Thus, the group differences are such that the S-curve patterns visible in Figure 5 are slightly steeper for the Leiden participants, and even more steep for the Ghent participants. However, as the [ε~εi] condition was a control condition, this does not matter all that much: these differences must be ascribed solely to the differences in the [εi] phone, which were already covered in a much more meaningful way in the [eː~εi] model. Rather, the [ε~εi] model serves to show that a classic S-curve pattern is obtained when two arbitrary sounds are morphed together in a rhyme-decision experiment, providing additional validation of the experimental method itself.

### 3.3 Discussion

Contrary to what would be expected based on the results from Experiment 1, Experiment 2 did not reveal significant differences at the level of the individual in the cluster analysis. The group-level analysis, however, showed the expected effects of “Step” and its interaction with “Following segment = non-/l/” for all three models.

For the [eː~εi] model, the results show that a following coda /l/ makes participants more likely to opt for the diphthong target as a function of the morphing step. In other words, a following coda /l/ shifts participants’ perceptual category boundary further towards /eː/. This is in line with the prediction that participants allow for some diphthongization to be present in an /eː/ realization, but only when it is not followed by coda /l/. This result shows that participants perceptually compensate for some diphthongization in the speech signal, but only in the phonological context where such diphthongization is allowed, demonstrating phonotactic knowledge. A second finding was that participants’ category boundaries are located at different positions. The Leiden group initially shows smaller odds of perceiving the diphthong target, but at later morphing steps catches up to the baseline, implying that the Leiden participants’ category boundary lies closer to /εi/. This agrees with the prediction that this participant group is willing to tolerate more diphthongization in the speech signal before switching from a slightly-diphthongized-/e:/ percept to a slightly-monophthongized-/εi/ percept. The same is true for the migrant participants, but only in the /l/ condition, showing that these participants compensate more strongly for diphthongization in a context in which it is unexpected, mirroring what they do in production. The [eː~εi] condition does not sample the *entire* continuum of possible realizations of /eː/ and /εi/, due to the on-going lowering of the latter diphthong. This is reflected in the results: even in the final step of the continuum, the proportion of /εi/ responses is still low. Compare this to the [oː~ɑu] condition, in which the proportion of diphthong responses is much higher in all four steps of the continuum. This condition sampled the *full* range of the diphthong phoneme, including the lowered [ɑu] realization, and for this reason reaches a much higher proportion of diphthong percepts at the final stage of the realization, which is 80% on the full [oː~ɑu] continuum. Note that sampling a wider continuum also implies using larger step sizes along the four intermediate points: the first step of 80% [oː] morphed with 20% [ɑu] includes more diphthongization than the first step of the [eː~εi] continuum would have. This is also reflected in the results: the proportion of diphthong responses at all four steps is higher for the [oː~ɑu] condition than it is for the [eː~εi] condition. The general trends of more diphthong responses at later morphing steps, and of more diphthong responses when there is a following coda /l/, are similar between the two conditions. Note, however, that the group differences are different: the statistical analysis revealed significant group differences for the [eː~εi] condition, but did not do so for the [oː~ɑu] condition. This suggests that the group differences are robust chiefly in the former half of the monophthong–diphthong continuum. As this is the part pertaining to the diphthongization of /eː,øː,oː/, which is a much older sound change (first mentioned by Zwaardemaker and Eijkman, 1924) than the lowering of the original diphthongs (of which the earliest reference in section 1.4 is Gussenhoven and Broeders, 1976), this result is not wholly surprising.

The [ε~εi] model served as a control condition; here, the different effects add up to produce a classic S-curve pattern, which is expected if two arbitrary sounds are morphed together. This curve has significantly sharper edges in the migrant group. This is likely to be due to the smaller sample size of this group, which provides less opportunity for sharper edges to be smoothed out by many observations.

## 4 Link between production and perception

The results from section 2.2.2 found significant inter-individual differences in their adoption of the sound changes in production, but the same individual variation was not found in perception, where only group-level results were found. Following Evans and Iverson (2007), however, it is possible that the individual results for the perception data are correlated with those for the production data. As explained in the Introduction, the existence of such a production–perception link is of major importance for the individual adoption and community propagation of sound change. Section 2.2.2 showed that the individual variation in production is represented well by the “Following segment = non-/l/” BLUPs; the present section investigates whether the variation in these BLUPs can be (partly) explained by the BLUPs from the individual-level analysis of the perception data.

### 4.1 Method

Running 24 correlation tests (3 models for the perception task × 8 random-effect vectors each) would be improper for reasons of multiple comparisons. However, since BLUPs are Gaussian random variables, it is possible to test each of these 24 correlations simultaneously by simply performing a linear regression analysis of these 106×24 data points onto the 106 BLUPs obtained from the analysis of the production experiment. Thus, a linear-regression analysis was performed with the “Following segment = non-/l/” BLUPs from the production experiment as the dependent variable, and the 24 sets of BLUPs from the rhyme-decision task as covariates. All variables included were standardized (i.e., z-transformed), so that the estimated regression coefficients are exactly equal to Pearson product-moment correlation coefficients. As comparisons *between* these 24 predictors are not of interest, neither an intercept term nor any interactions were included in the model. The R code for this analysis is available at https://figshare.com/s/731e0a32480e876530e0 as the file correlation.R.

### 4.2 Results

**Figure 7.**
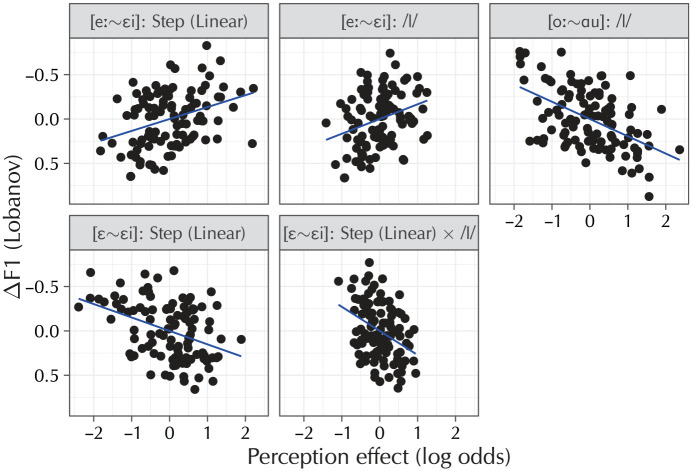
The partial correlations that reached significance in the analysis (*n* = 106), backtransformed to the original linear-predictor scales. Participants who produce more diphthongization are more likely to indicate a diphthong percept in the [eː~εi] perception model at later morphing steps as well as when a coda /l/ followed the vowel. In the [oː~ɑu] model, participants who produce more diphthongization are less likely to indicate a diphthong percept when a coda /l/ follows. Finally, in the control model [ε~εi], participants who diphthongize more in production are less likely to indicate a diphthong percept as a function of the morphing step in both the non-/l/ and /l/ conditions.

The results of the analysis are shown in Table 5; Figure 7 provides a visualization of the correlations that reached significance. These are partial-effect plots, meaning that the plots show the effect for each correlation term while controlling for the other 23 terms present in the linear-regression model. The standardization has been reverted in this figure, such that the visualized correlations are on the same scale as the original BLUPs and are therefore directly interpretable as relationships between the individual differences in DF1 in production and the log-odds of the diphthong percept in perception. In total and after adjusting for multiple testing, the individual differences in the perception data were able to account for 34% of the variance in the individual differences in the production data.

**Table 5.** Correlations of the various random slopes for the rhyme-decision task with the “Following segment = non-/l/” random slope from the production task (*n* = 106). *F*_(24, 82)_ = 3.23, p < .001, *R*^2^ = .49, *R*^2^adj = .34. The correlations are visualized in Figure 7.

Factor	Estimate (SE)	*t*	*p*	Sig.
Model = [eː~εi], Intercept	.24 (.13)	1.80	.08	
Model = [eː~εi], Step (Linear)	−.27 (.12)	−2.30	.02	*
Model = [eː~εi], Step (Quadratic)	.02 (.09)	0.16	.87	
Model = [eː~εi], Step (Cubic)	−.12 (.09)	−1.41	.16	
Model = [eː~εi], /l/	−.23 (.09)	−2.49	.01	*
Model = [eː~εi], Step (Linear) × /l/	−.08 (.10)	−0.77	.44	
Model = [eː~εi], Step (Quadratic) × /l/	.12 (.09)	1.40	.17	
Model = [eː~εi], Step (Cubic) × /l/	−.02 (.09)	−0.22	.83	
Model = [oː~ɑu], Intercept	.13 (.10)	1.33	.19	
Model = [oː~ɑu], Step (Linear)	.01 (.15)	0.08	.94	
Model = [oː~ɑu], Step (Quadratic)	−.17 (.11)	−1.54	.13	
Model = [oː~ɑu], Step (Cubic)	−.06 (.09)	−0.60	.55	
Model = [oː~ɑu], /l/	.28 (.11)	2.53	.01	*
Model = [oː~ɑu], Step (Linear) × /l/	.16 (.12)	1.36	.18	
Model = [oː~ɑu], Step (Quadratic) × /l/	.01 (.09)	0.12	.91	
Model = [oː~ɑu], Step (Cubic) × /l/	−.09 (.10)	−0.86	.39	
Model = [ε~εi], Intercept	−.12 (.13)	−0.91	.36	
Model = [ε~εi], Step (Linear)	.43 (.13)	3.31	< .01	**
Model = [ε~εi], Step (Quadratic)	−.02 (.10)	−0.20	.84	
Model = [ε~εi], Step (Cubic)	.02 (.10)	0.23	.82	
Model = [ε~εi], /l/	−.09 (.11)	−0.86	.39	
Model = [ε~εi], Step (Linear) × /l/	.31 (.11)	2.87	< .01	**
Model = [ε~εi], Step (Quadratic) × /l/	.08 (.11)	0.73	.46	
Model = [ε~εi], Step (Cubic) × /l/	.08 (.11)	0.77	.44	

Participants who diphthongized less strongly (higher DF1) in the production task were also less likely to indicate a diphthong percept in the [eː~εi] condition of the perception task (r = −.27). Similarly, if this condition in the perception experiment contained a following coda /l/, participants became more likely to indicate a diphthong percept if they produced more diphthongization themselves (r = −.23). In the [oː~ɑu] condition, this effect was reversed: participants were more likely to indicate a diphthong percept if they themselves produced *less* diphthongization (r = .28). Finally, in the [ε~εi] condition, participants who diphthongized less strongly in production were quicker to perceive a diphthong as a function of the morphing step (r = .43), and became so even more in the /l/ context (r = .31).

### 4.3 Discussion

The correlations show that participants who realize vowels such as /eː/ with less diphthongization are also less likely to perceive slightly diphthongized realizations of this vowel as realizations of /εi/. These participants are thus less advanced on the sound change diphthongizing [eː] to [ei]: they diphthongize less themselves, and perceptually allow for more diphthongization in the speech signal before switching their percept to the diphthongal target. For participants who are further along the sound changes, the presence of a following coda /l/ makes an important difference. In this situation, these participants have no reason to expect diphthongization based on the phonological context. This is why, at the group level, this condition resulted in significantly more diphthong percepts. For participants who are less far along on the sound change, that is, who diphthongize less in production, the difference a following coda /l/ makes is much smaller, as these participants have no need for the phonological rule blocking diphthongization before /l/. The [eː~εi] correlations also reflect this. These results corroborate the findings by Beddor et al. (2018) and Coetzee et al. (2018).

In the [oː~ɑu] condition of the perception experiment, the latter effect reverses. Recall that this is also the condition that sampled a more complete continuum of the diphthong phoneme, and the condition in which between-groups differences were not borne out. The latter suggests that the differences between /l/ and non-/l/ observed in this condition are not driven by between-groups differences in phonological rules. If participants do not assign differential weight to the effect of a following coda /l/ (as the previous paragraph argued for the [eː~εi] condition), the observed correlation follows naturally. Participants who are further along the sound changes then allow for more intrinsic diphthongization, and will thus indicate more monophthong percepts even in the presence of a following coda /l/, whereas participants who are less far along the sound changes are more likely to indicate a diphthong percept.

In the control condition [ε~εi], participants who diphthongize less in production are *more* likely to perceive slightly diphthongized realizations as /εi/. This condition is not affected by the on-going sound changes, and hence there is no reason for participants to expect any intrinsic diphthongization to be present in the monophthongal endpoint of the perception continuum. In this case, participants who produce less diphthongization also allow for less diphthongization in the speech signal before switching to an /εi/ percept, so this vowel’s category distinctions in perception and production directly mirror one another. Similarly, in the [eː~εi] condition when followed by coda /l/, participants also have no reason to expect any intrinsic diphthongization and indeed show the same behavior.

## 5 General discussion

The main goals of this paper were to further advance our understanding of sound change by investigating in detail a contact-driven phonological change and by also taking into account variation at the individual level. At the group level, both the production and the perception results showed significant influences of the distinction between a following coda /l/ versus another consonant as a function of the participant group, demonstrating between-groups differences in phonological knowledge. This was also borne out in a particularly clear way by the individual-level production results. While the group-level results in production simply placed the migrant group in between the two control groups, the individual-level results revealed a more nuanced picture, by showing that the migrant group was not homogeneous: some individuals had adapted so much that they were classified with the Leiden participants, but other individuals had not and were classified with the Ghent participants.

The results for the rhyme-decision task were quite different: at the group level, the migrant group showed a systematic shift in one of the two critical conditions (the boundary between [eː~εi]) and in the control condition (the boundary between [ε~εi]). This pattern of results suggests that the migrant participants’ perception of the /εi/ category shifted more towards the Netherlandic system. Contrary to the results for the production data, these findings were only observed at the level of the whole group; in the variation between individual participants, no systematic patterns were observed. However, significant and meaningful relationships were found between the individual differences in perception and those in production.

The production results and their correlation with the perception results corroborate the results by Evans and Iverson (2007), and also agree with findings from the field of L2 acquisition, which show that L2 learners change their production over long periods of time, but not their perception (Flege, 1987; Flege & Wayland, 2019). The production results and their substantial inter-individual differences are also in line with Nycz (2013) and Evans and Iverson (2007). Although Evans and Iverson (2007) do not actually discuss it, the production results in their Table 1 (p. 3817) show that some speakers changed their phonetic implementations to larger or smaller degrees, and some did not at all. When considered as a single group, their results show a small but systematic change across the board. The production findings from the present experiment paint exactly the same picture: some migrant individuals have changed their Flemish-Dutch vowels to conform to the Netherlandic-Dutch system, some have not, and at the group level, these individual effects are large enough to quantitatively push the whole group towards a more Netherlandic vowel system. The individual differences between perception and production fit right into the picture painted by Beddor et al. (2018) and Coetzee et al. (2018), in that participants who are more advanced in production are, generally speaking, also more advanced in perception. It additionally appeared that, while section 3’s results for the [eː~εi] condition could be explained by differences in phonological knowledge between the participants, the results for the [oː~ɑu] condition were driven more by phonetic expectations than by phonological knowledge. Following Baker et al. (2011), Pinget (2015), and Pinget et al. (2019), this is in line with the [oː~ɑu] data reflecting a sound change that is in an earlier stage of completion, in which phonetic variation has not yet been fully encoded into a complete sound change. As this continuum incorporates not just the diphthongization of the tense mid vowels but also the much more recent lowering of the original diphthongs, this is a possibility, although the present set of experiments cannot prove this conjecture.

On the question if adoption starts in perception or in production, the results from the present paper are in line with Evans and Iverson (2007): change in production was easily detected, change in perception was not. Specifically, while the sociolinguistic migrants as a group had shifted the category boundary of at least their /εi/ phoneme to be more like the Netherlandic group, it was not possible to single out a discrete set of specific individuals who were uniquely responsible for this group-level effect, although individual-level correlations between perception and production were found (which was also true for Evans and Iverson). These correspondences make it plausible that the changes in these socoiolinguistic migrants started out in production, and hence that the contact-driven phonological change studied here is wholly similar to Evans and Iverson’s (2007) contact-driven phonetic change. These results are compatible with the observation by Pinget (2015) and Pinget et al. (2019) that sound changes become production-driven when they have almost come to completion. This follows from the idea that sociolinguistic migrants are comparable to individuals who have remained conservative while their environment has adopted a novel sound change.

## 6 Conclusion

The present paper investigated the role of the individual in the adoption of sound change. The focus of investigation was sociolinguistic migrants, in this case Flemish-Dutch speakers who moved to the Netherlands multiple years to decades ago. The results are partially in line with the findings by Evans and Iverson (2007). On the one hand, in agreement with Evans and Iverson, group-level adoption of the sound changes was found in production; specifically, the group as a whole had undergone a quantitative shift to be more Netherlandic-Dutch-like, and 10 of the 18 participants had changed to such a degree that, in a cluster analysis, they were classified as having become qualitatively Netherlandic. The present study also found similar effects in perception in a group-level analysis, but the same effects were not borne out in an individual-level analysis of the perception data. However, individual-level correlations were found between perception and production. These results are in line with previous findings on individual differences by Beddor et al. (2018) and Coetzee et al. (2018). They also fall in line with findings on the individual level by Baker et al. (2011), Pinget (2015), and Pinget et al. (2019) inasmuch as they suggest that younger sound changes are more reliant on superficial phonetic variation than on structural phonological variation. Taken together, the results from the present study contribute to our knowledge of phonological change, and also provide another demonstration how individual differences can provide a richer view of the adoption of sound change than could have been obtained by considering only patterns at the level of the group, precisely as Stevens and Harrington (2014) had foreshadowed.

The present study is not without its limitations, of which I highlight one that could inspire future research. The migrant group of participants was quite small (n = 18), which limited the individual-level analyses reported in this paper. While the results were very clear for purposes of the present paper, in showing that the productions of the migrant group could be classified into the expected two groups with sufficient statistical power, the migrant group was too small to determine what factors drove this classification in the first place. For instance, do participants’ degrees of adoption correlate with the amount of time they have lived in the Netherlands? If it does, does it do so still after controlling for participant age—in other words, do the participants adopt lifespan changes or are these instances of age-grading (Wagner, 2012)? To further tease apart these two possibilities, it would be particularly fortuitous if future research recruited control participants matched in age to the migrant group. However, such evidence could also be gathered from other sources, such as the Dutch teacher corpus (van Hout et al., 1999), which maps the regional variation in the sound changes discussed in this paper in great detail, and in which age was explicitly taken into account as a variable during the data collection. The combination and integration of these different sources of knowledge into a single larger picture of the on-going Dutch sound changes would be a welcome continuation of the research presented in this paper.

## Figures and Tables

**Figure 8.**
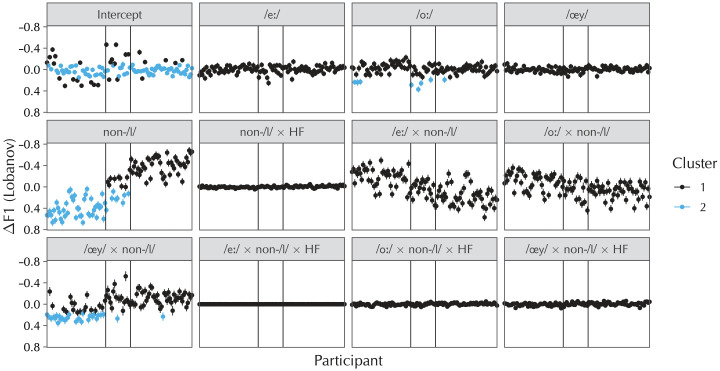
Separate BLUPs for the each of the random effects present in the by-individuals (*n* = 106) model for the production data, with separate cluster analyses for each panel. As in Figure 3, each panel is separated into three panes: the left pane shows the participants from the Ghent group, the middle pane shows the participants from the migrant group, and the right pane shows the participants from the Leiden group.

## Appendix

**Appendix A.** Word list for Experiment 1

achteruitkijkspiegel	bewegingsmogelijkheden	deugdzaamheid
adieu	bezopener	dialogen
afstotelijk	bijgepunt	dichtstbijzijnde
alleenstaand	blasfemie	doodstraf
auto-ongevallen	boerderij	dreun
autokerkhoven	bonis	dromen
autoritje	breakdancing	droogkamer
autosleutels	breedgeschouderde	droogshampoo
bakkerij	brekebeen	dweep
bedruipende	buidelrat	eindig
beenbeschermers	buil	eindresultaten
beenbreuken	buis	enigszins
beloninkje	buiswater	entertainer
bereid	buitenland	eureka
bereikbaar	buitenschools	evenwicht
bespeel	bullshit	existentieel
beu	bureaublad	familiariteit
beugel	cacao	filosoof
beul	cacaoboom	financieel
beult	degusteren	foyer
bevrijdingsactie	deug	fundamenteel
gebruiksmogelijkheden	hoog	kokosvlees
geelst	hoogmoed	komen
geestesleven	houder	koolblad
gefoezel	hout	koolsoep
geilaard	houtbewerker	koolzuurgas
geilheid	houthandelaren	koopziek
gelegenheden	houtig	kreek
gelouterde	houtindustrie	kwartaal
genereus	houtluis	kwartaalblad
geneugten	houtsnijder	kwijl
geul	houtsnijwerken	langszijde
geweigerd	houtwerker	leid
gonorroe	houweel	leidinggevende
goud	huichelaar	lepelaar
grootbrengen	huiskamerbijeenkomst	leugenaar
heethoofd	huisnummer	levendig
heil	Huisschilders	lijk
heildronk	iel	lijntrekkers
heul	ijlkoorts	lou
heulde	ijsbeer	louter
heult	immoreel	luistervinkte
heup	kaneel	makreel
heus	kannibaals	manipulatie
heuvel	kleuter	mantilla
hogelijk	kleverigheid	medisch
holocaust	kloostermuren	meegesleept
hoofdrol	knuisten	meegevoerd
meekwamen	ouderling	raap
meervoudige	ouderschapsverlof	rancuneus
meinedig	oudste	religieus
meineed	oudtante	reserveren
mitotisch	oudtantes	rijnstenen
morfologische	overgehaald	rijzige
necrofiel	overgestapt	rioolrat
nijlpaard	overgezonden	rioolstelsel
nijpender	overgrootvader	risico-kinderen
nobelere	peil	ritueel
noordpoolcirkel	peilsignaal	rovertje
officieus	peilstift	ruil
omgeving	peilstok	ruilbeurs
onderkoeld	personeelsbestand	ruilhandel
ondersteun	personeelsgegevens	ruilmiddel
onophoudelijk	personenwagen	saus
onpeilbaar	pieptoon	schadelijk
ontboden	pijlinktvis	schapenfarm
ontleedtafel	pijlsnel	schoolagenda
ontsteking	pijn	schoolfrik
onverdeeld	pijpkruid	schoolgebied
onverwijld	pistoolgreep	schooljuffrouw
oostelijk	polio	schoolkameraad
oosterse	pruikje	schoolkamp
ouden	pruil	schoolkleuren
ouderdomsproces	pruimentaart	schoolreis
ouderlijk	puilde	schoolteam
schoolvoorstelling	stuiter	uithoren
schoolvriend	stuiver	uitladen
schoolziek	subtiel	uitnamen
schroothoop	suikerraffinaderij	uitpuilend
schuiladres	suikerziek	uitreiking
schuilgaan	tegenstrijdig	uitscheld
schuilgehouden	tegenvoeter	uitspraak
schuilhut	terpentine	uitzwaaien
schuilkelder	terwijl	veiligheidsredenen
schuilnaam	textielindustrie	veiligheidsscheermes
schuilplaats	thuishoorden	veiligheidssituatie
sein	thuismarkt	verbeid
smeul	tijdregistratie	verdraaglijk
smeult	tijdschema	verdrievoudigd
smoel	tijdsprobleem	vereenzaamd
speelbal	toegespitst	vereisen
speelbare	toveraar	vergeet
speelgoedwinkel	uil	vergeving
speelhal	uilskuiken	verhouding
speelkaart	uitbarsten	verijs
speelmakker	uitbazuinen	verkoelde
speelster	uitbuiten	vermeende
staal	uitdrukking	verpauperd
staalplaat	uitgehuwelijkt	verpersoonlijk
stoomstrijkijzers	uitgekeken	verruil
strategen	uitgerekend	verschuil
stuiptrekkende	uitgroeide	verstuikt
vertonen	wijdvertakte	zeult
vervloek	wijnhuis	zintuiglijk
vervuil	wijnkleurige	zodanig
verzeild	wijziging	zoem
verzwijg	woonark	zotternij
vleesetende	zedelijkheidsgevoel	zouden
vleeshouwer	zeilt	zoutarm
vleugel	zeiltocht	zoutjes
vloekwoord	zeilwedstrijd	zoutwinning
waarheidlievend	zeug	zuil
wede	zeul	zwoel
wezenfonds	zeulde	

### Appendix B. Full BLUPs for Experiment 1

**Appendix C.** Prime–target list for Experiment 2

bauke – zauke	blelder – zijlder	debe – webe
bauke – zoke	blene – hene	debe – wijbe
bebbe – webbe	blene – hijne	deter – zeter
bebbe – wijbe	blete – lete	deter – zijter
begge – wegge	blete – lijte	devver – levver
begge – wijge	bleter – leter	devver – lijver
bekker – hekker	bleter – lijter	drever – rever
bekker – hijker	brauver – vauver	drever – rijver
bemme – zemme	brauver – vover	dweelder – bleelder
bemme – zijme	bredde – stedde	dweelder – blijlder
benne – henne	bredde – stijde	dwever – krever
benne – hijne	bredder – tedder	dwever – krijver
bezze – hezze	bredder – tijder	fauver – jauver
bezze – hijze	breelder – veelder	fauver – jover
blaude – graude	breelder – vijlder	fedde – sedde
blaude – grode	brette – tette	fedde – sijde
blaulder – waulder	brette – tijte	fedder – kedder
blaulder – woolder	bretter – letter	fedder – kijder
blauver – schauver	bretter – lijter	fevver – wevver
blauver – schover	brever – tever	fevver – wijver
blebe – zebe	brever – tijver	flaulde – maulde
blebe – zijbe	bwelde – slelde	flaulde – moolde
blege – bege	bwelde – slijlde	flaulder – braulder
blege – bijge	dauver – trauver	flaulder – broolder
blelder – zelder	dauver – trover	fleelder – seelder
fleelder – sijlder	jauze – nauze	kreder – dijder
flelder – selder	jauze – noze	kreelder – feelder
flelder – sijlder	jede – krede	kreelder – fijlder
gauver – nauver	jede – krijde	krelder – nelder
gauver – nover	jevver – sevver	krelder – nijlder
gleelde – kreelde	jevver – sijver	kwaulde – gaulde
gleelde – krijlde	kebe – tebe	kwaulde – goolde
glelde – flelde	kebe – tijbe	kwaulder – fraulder
glelde – flijlde	keder – zeder	kwaulder – froolder
greelde – treelde	keder – zijder	kweelder – zeelder
greelde – trijlde	kegge – begge	kweelder – zijlder
greelder – teelder	kegge – bijge	lauder – zauder
greelder – tijlder	klaulde – faulde	lauder – zoder
grelde – trelde	klaulde – foolde	lebbe – zebbe
grelde – trijlde	klaulder – laulder	lebbe – zijbe
grelder – telder	klaulder – loolder	mauder – jauder
grelder – tijlder	klauver – vrauver	mauder – joder
grevver – revver	klauver – vrover	meker – heker
grevver – rijver	kleelder – geelder	meker – hijker
gweelde – fleelde	kleelder – gijlder	nauve – zauve
gweelde – flijlde	klelder – kwelder	nauve – zove
jaube – haube	klelder – kwijlder	nebe – bebe
jaube – hobe	kneelde – pleelde	nebe – bijbe
jauge – nauge	kneelde – plijlde	neke – heke
jauge – noge	kraulder – naulder	neke – hijke
jauter – nauter	kraulder – noolder	nete – tete
jauter – noter	kreder – deder	nete – tijte
neter – teter	prever – twijver	sjeelde – vreelde
neter – tijter	psaulder – draulder	sjeelde – vrijlde
pauder – bauder	psaulder – droolder	skeelde – freelde
pauder – boder	pselde – lelde	skeelde – frijlde
pevver – tevver	pselde – lijlde	skelde – twelde
pevver – tijver	rebe – hebe	skelde – twijlde
pfelde – klelde	rebe – hijbe	skelder – twelder
pfelde – klijlde	sauver – bauver	skelder – twijlder
pjelde – xelde	sauver – bover	slaulde – baulde
pjelde – xijlde	schaube – kaube	slaulde – boolde
plaulde – vaulde	schaube – kobe	slaulder – taulder
plaulde – voolde	schauge – kauge	slaulder – toolder
plaulder – praulder	schauge – koge	sleelder – peelder
plaulder – proolder	schauke – nauke	sleelder – pijlder
plauver – mauver	schauke – noke	slelder – dwelder
plauver – mover	schaulder – jaulder	slelder – dwijlder
pleelder – jeelder	schaulder – joolder	smeelde – bleelde
pleelder – jijlder	schevver – zevver	smeelde – blijlde
plelder – jelder	schevver – zijver	smelde – drelde
plelder – jijlder	sedder – zedder	smelde – drijlde
plever – pever	sedder – zijder	smelder – drelder
plever – pijver	sfaulde – praulde	smelder – drijlder
preelder – steelder	sfaulde – proolde	snaulde – draulde
preelder – stijlder	sfeelde – preelde	snaulde – droolde
prelder – stelder	sfeelde – prijlde	snaulder – graulder
prelder – stijlder	sfelde – blelde	snaulder – groolder
prever – twever	sfelde – blijlde	sneelde – sleelde
sneelde – slijlde	srelde – grelde	sweelder – drijlder
snever – jever	srelde – grijlde	swelde – delde
snever – jijver	staude – gaude	swelde – dijlde
spaulde – naulde	staude – gode	swelder – relder
spaulde – noolde	staulde – jaulde	swelder – rijlder
spaulder – gaulder	staulde – joolde	taude – paude
spaulder – goolder	staulder – raulder	taude – pode
speelder – leelder	staulder – roolder	tegge – wegge
speelder – lijlder	stauver – lauver	tegge – wijge
spever – dever	stauver – lover	tetter – hetter
spever – dijver	stedder – hedder	tetter – hijter
spleelde – kleelde	stedder – hijder	tezze – zezze
spleelde – klijlde	stetter – wetter	tezze – zijze
splelde – krelde	stetter – wijter	tjaulder – baulder
splelde – krijlde	straulde – fraulde	tjaulder – boolder
spraulde – braulde	straulde – froolde	tjeelde – dreelde
spraulde – broolde	strelde – frelde	tjeelde – drijlde
spreelde – breelde	strelde – frijlde	tjelde – jelde
spreelde – brijlde	strelder – frelder	tjelde – jijlde
sprelde – brelde	strelder – frijlder	trauder – nauder
sprelde – brijlde	strevver – mevver	trauder – noder
sprever – mever	strevver – mijver	trauge – kauge
sprever – mijver	swaulde – kraulde	trauge – koge
sraulde – xaulde	swaulde – kroolde	traute – jaute
sraulde – xoolde	sweelde – leelde	traute – jote
sreelde – greelde	sweelde – lijlde	treelder – neelder
sreelde – grijlde	sweelder – dreelder	treelder – nijlder
trelder – belder	vedde – kedde	wauver – kauver
trelder – bijlder	vedde – kijde	wauver – kover
trever – sever	vedder – ledder	weme – heme
trever – sijver	vedder – lijder	weme – hijme
twaulde – zaulde	vede – kede	wemme – hemme
twaulde – zoolde	vede – kijde	wemme – hijme
twaulder – maulder	vleelde – tweelde	weppe – heppe
twaulder – moolder	vleelde – twijlde	weppe – hijpe
twebe – lebe	vlelde – prelde	xaulder – saulder
twebe – lijbe	vlelde – prijlde	xaulder – soolder
tweder – heder	vrauder – vauder	xelder – pelder
tweder – hijder	vrauder – voder	xelder – pijlder
tweelder – beelder	vraulde – waulde	zevve – hevve
tweelder – bijlder	vraulde – woolde	zevve – hijve
tweke – meke	vraulder – vaulder	zwaulde – graulde
tweke – mijke	vraulder – voolder	zwaulde – groolde
vaube – naube	vrebe – mebe	zweelde – jeelde
vaube – nobe	vrebe – mijbe	zweelde – jijlde
vauge – nauge	vreelder – deelder	zweelder – reelder
vauge – noge	vreelder – dijlder	zweelder – rijlder
vauke – hauke	vrelder – delder	zwelder – lelder
vauke – hoke	vrelder – dijlder	zwelder – lijlder

**Appendix D.** Full results of Experiment 2

Factor	Estimate (SE)	Odds ratio	*z*	*p*	Sig.
**Model = [eː~ɛi]**					
Intercept	−1.46 (0.10)	1 : 4.30	−14.49	< .001	***
Step (Linear)	1.24 (0.13)	3.47 : 1	9.87	< .001	***
Step (Quadratic)	0.33 (0.08)	1.39 : 1	4.22	< .001	***
Step (Cubic)	0.03 (0.08)	1.03 : 1	0.34	.74	
Following segment = /l/	0.83 (0.11)	2.29 : 1	7.59	< .001	***
Group = Migrant–Ghent	−0.51 (0.13)	1 : 1.66	−3.95	< .001	***
Group = Leiden–Others	0.07 (0.06)	1.07 : 1	1.06	.29	
Step (Linear) × /l/	1.68 (0.21)	5.37 : 1	8.14	< .001	***
Step (Quadratic) × /l/	−0.15 (0.14)	1 : 1.16	−1.07	.29	
Step (Cubic) × /l/	−0.13 (0.14)	1 : 1.14	−0.92	.36	
Step (Linear) × Migrant–Ghent	0.63 (0.15)	1.87 : 1	4.06	< .001	***
Step (Quadratic) × Migrant–Ghent	−0.04 (0.08)	1 : 1.04	−0.47	.64	
Step (Cubic) × Migrant–Ghent	0.05 (0.07)	1.05 : 1	0.66	.51	
Step (Linear) × Leiden–Others	0.01 (0.07)	1.01 : 1	0.17	.87	
Step (Quadratic) × Leiden–Others	0.04 (0.04)	1.04 : 1	1.11	.27	
Step (Cubic) × Leiden–Others	−0.05 (0.03)	1 : 1.05	−1.49	.14	
Following segment = /l/ × Migrant–Ghent	0.26 (0.13)	1.30 : 1	2.05	.04	
Following segment = /l/ × Leiden–Others	0.08 (0.06)	1.08 : 1	1.31	.19	
**Model = [oː~ɑu]**					
Intercept	0.20 (0.07)	1.22 : 1	2.76	.01	*
Step (Linear)	1.73 (0.15)	5.65 : 1	11.48	< .001	***
Step (Quadratic)	0.45 (0.11)	1.57 : 1	4.17	< .001	***
Step (Cubic)	−0.03 (0.11)	1 : 1.03	−0.28	.78	
Following segment = /l/	0.95 (0.12)	2.59 : 1	7.83	< .001	***
Step (Linear) × /l/	−0.66 (0.25)	1 : 1.93	−2.67	.01	*
Step (Quadratic) × /l/	−0.74 (0.22)	1 : 2.09	−3.32	< .001	**
Step (Cubic) × /l/	−0.19 (0.22)	1 : 1.21	−0.88	.38	
**Model = [ɛ~ɛi]**					
Intercept	−0.94 (0.07)	1 : 2.55	−13.12	< .001	***
Step (Linear)	2.49 (0.15)	12.06 : 1	16.44	< .001	***
Step (Quadratic)	0.27 (0.10)	1.31 : 1	2.59	.01	*
Step (Cubic)	−0.74 (0.11)	1 : 2.09	−6.99	< .001	***
Following segment = /l/	0.96 (0.12)	2.60 : 1	7.90	< .001	***
Group = Migrant–Ghent	−0.21 (0.08)	1 : 1.23	−2.64	.01	*
Group = Leiden–Others	0.09 (0.04)	1.10 : 1	2.50	.01	*
Step (Linear) × /l/	−1.38 (0.24)	1 : 3.98	−5.84	< .001	***
Step (Quadratic) × /l/	0.25 (0.21)	1.29 : 1	1.22	.22	
Step (Cubic) × /l/	0.87 (0.21)	2.38 : 1	4.13	< .001	***
Step (Linear) × Migrant–Ghent	0.43 (0.17)	1.53 : 1	2.52	.01	*
Step (Quadratic) × Migrant–Ghent	0.09 (0.09)	1.09 : 1	0.99	.32	
Step (Cubic) × Migrant–Ghent	−0.20 (0.09)	1 : 1.22	−2.25	.02	
Step (Linear) × Leiden–Others	−0.23 (0.08)	1 : 1.26	−2.91	< .01	*
Step (Quadratic) × Leiden–Others	−0.03 (0.04)	1 : 1.03	−0.67	.50	
Step (Cubic) × Leiden–Others	0.04 (0.04)	1.04 : 1	1.08	.28	
Following segment = /l/ × Migrant–Ghent	0.11 (0.12)	1.12 : 1	0.92	.36	
Following segment = /l/ × Leiden–Others	0.01 (0.06)	1.01 : 1	0.15	.88	
Step (Linear) × /l/ × Migrant–Ghent	−0.24 (0.23)	1 : 1.27	−1.04	.30	
Step (Quadratic) × /l/ × Migrant–Ghent	0.28 (0.18)	1.32 : 1	1.57	.12	
Step (Cubic) × /l/ × Migrant–Ghent	0.39 (0.18)	1.47 : 1	2.15	.03	
Step (Linear) × /l/ × Leiden–Others	−0.31 (0.11)	1 : 1.36	−2.95	< .01	**
Step (Quadratic) × /l/ × Leiden–Others	−0.18 (0.08)	1 : 1.19	−2.26	.02	
Step (Cubic) × /l/ × Leiden–Others	−0.09 (0.08)	1 : 1.10	−1.14	.25	

## Notes

1. I thank an anonymous reviewer for pointing out this possible dependency.

2. Five participants attended high school in non-Randstad areas, but as the results show, they were not significantly different from the participants who never left the Randstad after their childhood years.

3. CELEX was used as a starting point because it has phonetic transcriptions, but SUBTLEX was used for its more reliable indication of word frequency (Keuleers, Brysbaert, & New, 2010).

4. The additional data loss compared to the previous 94.7% is because if either element of a pair of <25%,75%> measures had been removed, their difference is undefined even if the other element of the pair was still present.

5. Likelihood-ratio tests are not applicable to bam models with non-Gaussian errors, as these are fitted using penalized quasi-likelihood (PQL); see Wood (2017, pp. 149–151) for details.

6. While Nycz (2013) does not report participants’ ages of arrival, Table 2.1 in Nycz (2011) shows that they ranged from 21 to 47 years of age, distributed approximately normally with M = 32.26 years and SD = 6.51 years.

7. The reason for fitting three separate models as opposed to one model with “Condition” as a factor is that the three models differ not only on the initial and final endpoints of the continuum, but also on the acoustic range spanned by the four intermediary steps. As such, quantitative differences between the three conditions are not interpretable: they could be due to linguistic differences or due to differences in the acoustic endpoints.
